# Sensor-Less Control of Mirror Manipulator Using Shape Memory Polyimide Composite Actuator: Experimental Work

**DOI:** 10.3390/s24123910

**Published:** 2024-06-17

**Authors:** Vetriselvi Velusamy, Dhanalakshmi Kaliaperumal, Seung-Bok Choi

**Affiliations:** 1 Department of Instrumentation and Control Engineering, National Institute of Technology, Tiruchirappalli 620015, India; vetriselvi.mv@gmail.com; 2 Department of Mechanical Engineering, The State University of New York, Korea (SUNY Korea), Incheon 21985, Republic of Korea; 3 Department of Mechanical Engineering, Industrial University of Ho Chi Minh City (IUH), Ho Chi Minh City 70000, Vietnam

**Keywords:** shape memory alloy, self-sensing, mirror manipulator, shape memory polyimide composite actuator, proportional derivative combined variable structure controller

## Abstract

Integrated thin film-based shape memory polyimide composites (SMPICs) are potentially attractive for efficient and compact design, thereby offering cost-effective applications. The objective of this article is to design and evaluate a mirror manipulator using an SMPIC as an actuator and a sensor with control. A sensor-less control strategy using the SMPIC (a self-sensing actuator) with a proportional derivative combined variable structure controller (PD-VSC) is proposed for position control of the mirror in both the vertical and angular directions. The mirror manipulator is able to move the mirror in the vertical and angular directions by 3.39 mm and 10.5 deg, respectively. A desired fast response is obtained as the performance under control. In addition, some benefits from the proposed control realization include good tracking, stable switching, no overshoot, no steady state oscillations, and robust disturbance rejection. These superior properties are experimentally validated to reflect practical feasibility.

## 1. Introduction

Mirror manipulation is a key function in a variety of industrial and commercial applications. For example, smart material-based actuators for mirror positioning are popular in medical imaging, interferometer systems, and laser beam steering due to their small size, reduced weight, low power consumption, and cost efficiency. The driving technology is the key feature for the performance and size of the mirror manipulator. The shape memory alloy (SMA) actuator-based driving technology is continuously advancing due to its remarkable properties of large recoverable deformation, high work density, biocompatibility, low actuation voltage, and high stress; eventually, SMAs in the form of thin film has received great importance in the field of research for their utilization as actuators and in sensing [1,2]. The fabrication of thin film SMAs on flexible substrates in the form of composites paves the way for their utility in micro systems for medical, robotics, and aerospace applications [3,4,5,6]. In comparison to the bare thin film SMA, shape memory alloy composites based on a flexible polyimide substrate exhibit larger displacement, do not require postprocess training, and require no bias and hence no structural complexities [7,8,9]. As a result, these composites can be used for developing low-cost and efficient actuators or sensors for many high-temperature (above 200 °C) applications. Various compositions of shape memory polyimide composites have been reported in the literature with respect to their design parameters, actuation parameters, and applications. Akira Ishida et al. developed the shape memory polyimide composite (SMPIC) actuator, in which they worked immensely on the sputter deposition of NiTi SMAs on polyimide substrates and explored their repeatable shape memory effect through joule heating. Ishida et al. [10] developed the NiTiCu SMPIC actuator and proved that it can move up and down like a dragonfly toy. Kishi Y. et al. [11] explored the cyclic behavior of an NiTi SMPIC actuator, and Kotnur et al. [12,13] developed an NiTi SMA/Polyimide composite actuator through sputtering at low temperatures, and they analyzed the influence of different parameters. Several researchers [14,15] reported that CuAlNi is an efficient and cost-efficient alternative to the NiTi SMA/Polyimide actuator. But, the major issue with the CuAlNi SMA is its poor ductility, which creates a reduction in its cyclic behavior.

Akash K. et al. [16] have overcome the issue of cyclic behavior by depositing the CuAlNi SMA on a prestrained flexible substrate, which induces the stress for cyclic behavior. They reported the fabrication of a CuAlNi SMA/Kapton polyimide composite using a thermal evaporation technique. The developed shape memory polyimide composite showed excellent adhesion of the SMA on the polyimide substrate. The scotch tape analysis has been used to test adhesion between the SMA and polyimide; also, they have explored the functional capabilities and extensively analyzed the electro-thermo-mechanical characteristics of the SMA/Polyimide composite actuator for different substrate thicknesses. In addition, this actuator can be applicable to several industrial applications such as circuit breakers, sensors for transformer oil temperature sensing, and microflapper at different substrate temperatures [16,17,18]. To improve the thermomechanical behavior and life cycle of the SMPIC actuator, manganese (Mn) has been added to the CuAlNi alloy, and the thermomechanical behavior of CuAlNiMn/Kapton composites was extensively studied by Akash K. and Jayachandran S. et al. [19,20] have developed four different SMPICs by depositing NiTi, NiTiCu, CuAlNi, and CuAlNiMn on a polyimide substrate using a flash evaporation technique, and its actuation characteristics, thermomechanical behavior, and lifecycle performances were compared by varying the input voltage, frequency, and load; they stated that the CuAlNiMn-based composite had higher displacement under loading conditions. Specifically, it can work for more than 24,000 cycles without significant loss.

A SMPIC actuator requires no bias; hence, no structural complexities and no postprocess training are required as well. The SMPIC is the best option to develop low-cost and efficient devices involving simple design. Hence, it is appropriate to be used as a smart actuator in mirror positioning applications to explore the potential of the SMPIC as an actuator cum sensor with control. This is considered as a study beyond the known state of the art.

To implement the closed-loop control of mirror manipulation tasks, external sensors are used to perform fast and accurate control objectives. Unfortunately, external sensors are not preferable for smart actuator applications because of their occupying space, size, cost, performances, measurement limitations, etc. Hence, the best alternative for this is self-sensing (sensing and actuation in the same device). Specifically in SMA, variations of the electrical resistance is sensitive to the strain, temperature, and structure. Hence, the displacement of the SMA actuator can be obtained by capturing the variations in electrical resistance during actuation using the self-sensing method [21,22,23,24]. To the best of the authors’ knowledge, the self-sensing closed-loop control scheme has not yet been proposed for an integrated SMPIC actuator-driven mirror positioning application. Consequently, the main technical contribution of this work is to experimentally investigate the advantages of an SMPIC actuator associated with a proper controller in which the feedback signals are achieved using the self-sensing method. To achieve the goal of this work, the mirror manipulator system is firstly designed by stating a working principle. As a next step, the actuation method for control of the mirror manipulator is discussed, followed by the formulation of the system model via the estimation of the motions of the mirror manipulator such as displacement. Subsequently, a controller to be applied to the mirror manipulator for the position control is designed by adopting a proportional derivative controller, which is combined with variable structure controller (PD-VSC). Then, the proposed control system is experimentally implemented, and several results such as tracking performance and disturbance rejection are obtained and compared with those acquired from the open-loop control. In comparative discussion, the benefits of the proposed control system mentioned in the above are considered all in terms of both the qualitatively and quantitatively, followed by the conclusion.

## 2. Design and Working Principle of the Mirror Manipulator

### 2.1. Schematic and Working Principle

**Figure 1:**
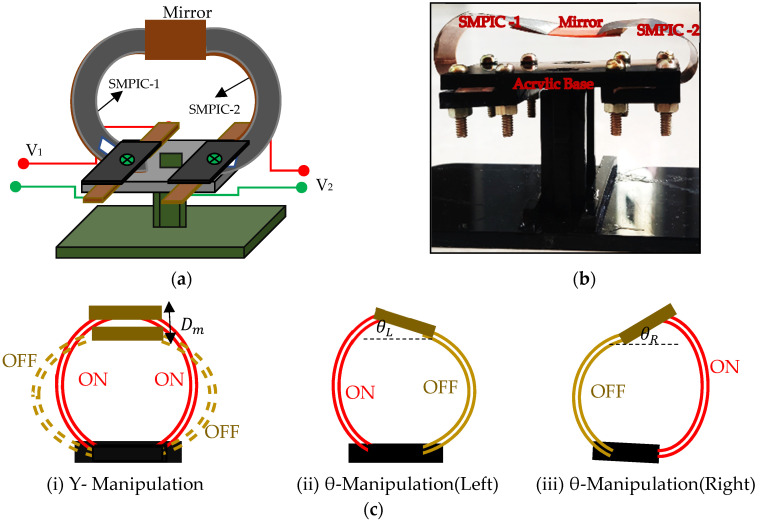
Schematic configuration and on–off actuating of the mirror manipulator. (**a**) Schematic configuration. (**b**) Photograph. (**c**) Three actuation methods.

A mechanical platform was designed using acrylic sheets to facilitate heating with provision to hold a couple of SMPICs. Two SMPICs were developed through the direct thermal evaporation method using CuAlNiMn that was coated on flexible, prestrained (curved), 75 μm thick Kapton polyimide HN sheets of the required dimension at an appropriate substrate temperature (150 °C). The developed actuators were bent upward due to cup-shaped training of the Kapton polyimide substrate, which displays a two-way shape memory effect without any postprocessing and training. One end of the SMPICs was cut in a two-leg (bileg) shape to facilitate joule heating. The two legs of both SMPICs were held on opposite sides of the acrylic base. Copper leads were provided to each leg of both SMPICs to minimize contact resistance and help current flow to the conductive SMA layer. A mirror of 60 mg weight was connected to the free end of both SMPICs. The schematic design, photograph, and actuation configuration of the proposed mirror manipulator are shown in Figure 1a–c, respectively.

The SMPICs were activated by applying voltage on the pair of copper leads, which was directly in contact with the conductive SMA layer using the joule heating method. Depending on the amount of electrical current passing through the conductive SMA layer, the position of the mirror changed in the out-of-plane motion due to the deflection (unfold motion) of the SMPICs. When voltage was removed, the SMPICs returned to their normal state due to the bias force of the trained polyimide sheet. The manipulator could move the mirror in both the vertical (Y manipulation) and angular (θ manipulation) directions based on the actuate configuration of both SMPICs. The mirror could move in the vertical and angular directions depending on the three types of actuating configurations. One type is for vertical displacement, and two types are for angular displacement. In the first type, both of the SMPICs were activated simultaneously; hence, the mirror moved vertically ($D_{m})$. In the second and third types, either of the SMPICs was actuated to move the mirror in the angular directions, i.e., SMPIC-1 was actuated and SMPIC-2 was not, to move the mirror toward the left angle direction ($\theta_{L}$). Similarly, SMPIC-2 was activated and SMPIC-1 is not to move the mirror toward a right angle direction ($\theta_{R}$). The dimensions of the SMPICs and the mirror are given in Table 1.

### 2.2. Actuator of the Mirror Manipulator

**Figure 2:**
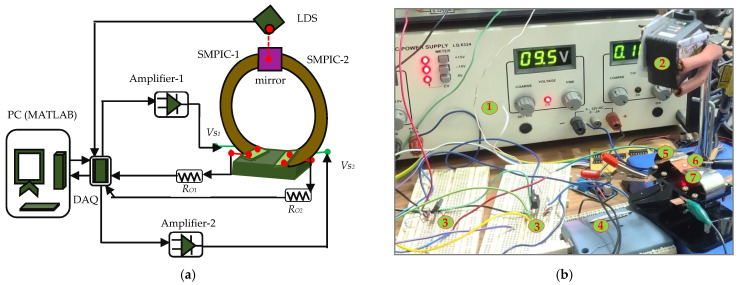
Experimental setup to actuate the mirror manipulator. (**a**) Schematic representation. (**b**) Photograph: 1. Power supply, 2. Laser Displacement Sensor, 3. Amplifier circuits, 4. DAQ, 5. SMPIC-1, 6. Mirror, and 7. SMPIC-2.

**Figure 3:**
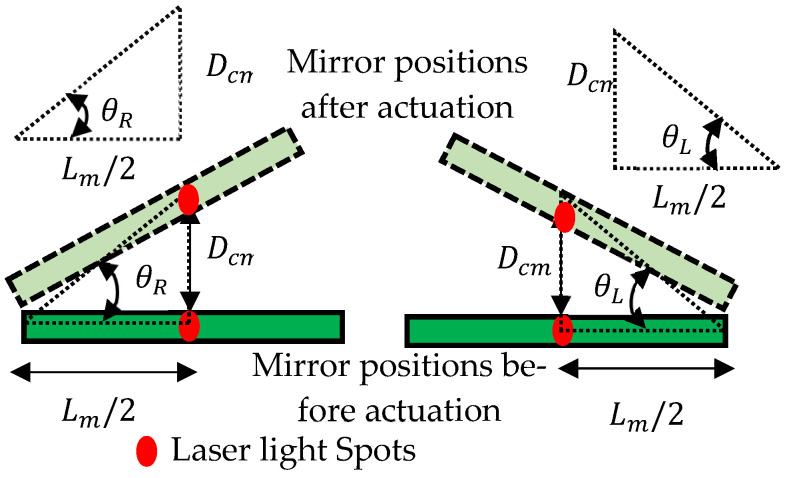
Estimation scheme of angular displacement.

The schematic representation and photograph of the experimental setup to actuate the mirror manipulator are shown in Figure 2a,b respectively. To activate the SMPIC-1 voltage signal, *Vs*_1_ was given through a USB-1408FS-Plus DAQ and an amplifier circuit-1 (OPA547). To activate the SMPIC-2 voltage signal, *Vs*_2_ was given through a USB-1408FS-Plus DAQ and an amplifier circuit-2 (OPA547). Both of the SMPICs were heated and cooled depending on the amplitude levels of the given voltage signals. A noncontact-type Keyence IL 100 laser displacement sensor (LDS) was held above the mirror and pointed at the center of the mirror, and it was used to measure the vertical displacement of the mirror. Data were collected in the PC through a DAQ. For the angular displacement, both the angles $\theta_{L}\;$and $\theta_{R}\;$were estimated from the vertical displacement of the mirror’s center point $(D_{cm)}\;$and measured using a laser displacement sensor and structural parameter of length $L_{m}$ of the mirror using the Equation (1). Figure 3 shows the angular displacement estimation logic and derived equation for the angular displacement in shown in Equation (1).

(1) $$\theta_{R}=\theta_{L}={tan}^{-1}(\frac{D_{cm}}{\frac{L_{m}}{2}})$$

**Figure 4:**
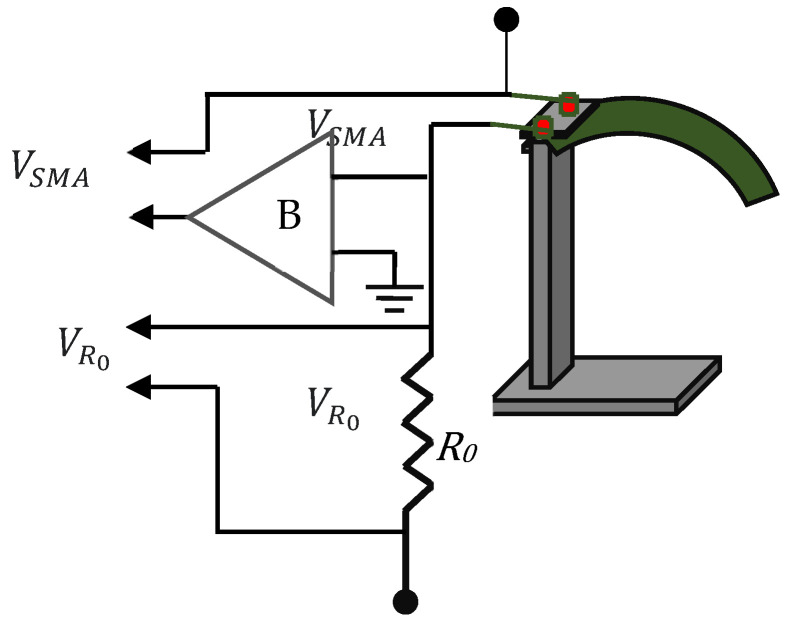
Resistance measurement circuit.

An external resistor $R_{O1}$ was connected in series to SMPIC-1, and $R_{O2}$ was connected in series to SMPIC-2. The voltage across the SMPIC-1 $V_{SO1}$ and the external resistance $V_{RO1}$ were used to evaluate the resistance variation of the SMPIC-1 using Equation (2). The voltage across the SMPIC-2 $V_{SO2}$ and the external resistance $V_{RO2}$ were used to evaluate the resistance variation of SMPIC-2 using Equation (3). Figure 4 shows the resistance measurement logic. The equations for the resistance estimation are given by

(2) $$R_{S1}=\frac{V_{SO1}}{V_{RO1}}.R_{O1}$$

(3) $$R_{S2}=\frac{V_{SO2}}{V_{RO2}}.R_{O2}$$

where, $\theta_{L}$ is the angular displacement of the mirror in the left angle direction in deg; $\theta_{R}$ is the angular displacement of the mirror in the right angle direction in deg; $D_{cm}$ is the vertical displacement of the mirror center point in mm; $L_{m}$ is the length of the mirror in mm; $R_{S1}$ is the resistance of SMPIC-1 in ohms; $R_{O1}$ is the external resistor connected in series to SMPIC-1 in ohms; $V_{SO1}$ is the voltage across SMPIC-1 in volts; $V_{RO1}$ is the voltage across the external resistor $R_{O1}$ in volts; $R_{S2}$ is the resistance of SMPIC-2 in ohms; $R_{O2}$ is the external resistor connected in series to SMPIC-1 in ohms; $V_{SO2}$ is the voltage across SMPIC-2 in volts; and $V_{RO2}$ is the voltage across external resistor $R_{O2}$ in volts.

Experiments were conducted to actuate the mirror manipulator in the vertical and angular directions. The open-loop performances of the manipulator for different input voltages up to 3 V and frequencies (1/20, 1/10, 1/5, 1/3, and 1/2 Hz frequencies with 3 V amplitude) were evaluated using the experimental setup as shown in Figure 3, and the results are listed in Table 2 and Table 3. The findings proved that the designed mirror manipulator could move the mirror 3.4 mm vertically and 10.8 deg angularly for a maximum input voltage of 3 V. The following operating conditions were observed for the efficient use of the mirror manipulator: a maximum vertical displacement of 3.39 mm was obtained to an operating frequency of 0.05 Hz, and a displacement of 0.99 mm was obtained for a maximum frequency of 0.5 Hz. Likewise, a maximum angular displacement of 9.7 deg was obtained to an operating frequency of 0.05 Hz, and a displacement of 2.6 deg was obtained for a maximum frequency of 0.5 Hz.

## 3. System Identification and Displacement Estimation

**Figure 5:**
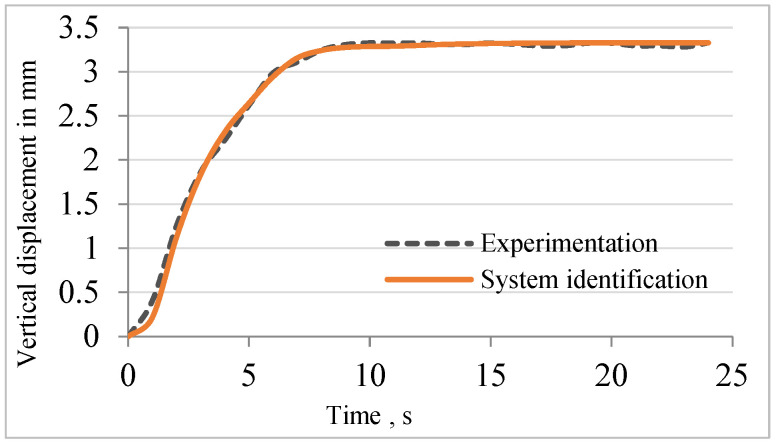
Validation of system identification.

Using the experimental data obtained from the open-loop test, a second-order transfer function with time delay was developed to relate the input voltage to the vertical displacement of the mirror. Equation (4) provides the transfer function between the vertical displacement of the mirror and the input voltage. Figure 5 shows the responses for the system identification and experimentation. The Final Prediction Error (FPE), Mean Square Error (MSE), and Fit Percentage of System Identification came out to 0.00022, 0.00016, and 97.09, respectively.

(4) $$G_{VD}=\frac{Vertical\;Displacement}{input\;voltage}=\frac{99.594{.e}^{\left(-0.05s\right)}}{s^{2}+255.1s+90.6}$$

(5) $$G_{AD}=\frac{Angular\;Displacement}{input\;voltage}={tan}^{-1}\frac{\frac{1}{2}\times (G_{VD})}{L/2}\times \frac{180}{PI}$$

Equation (5) gives the relationship between the vertical displacement and the angular displacement of the mirror, i.e., the equation was used to estimate angular displacement using vertical displacement of the mirror.

**Figure 6:**
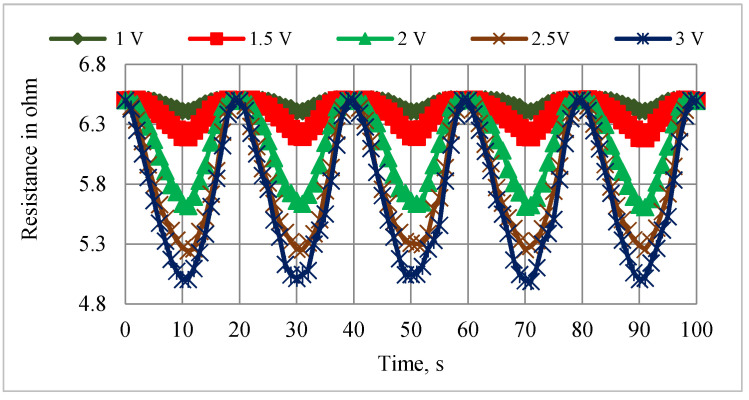
Resistance variation for various inputs.

**Figure 7:**
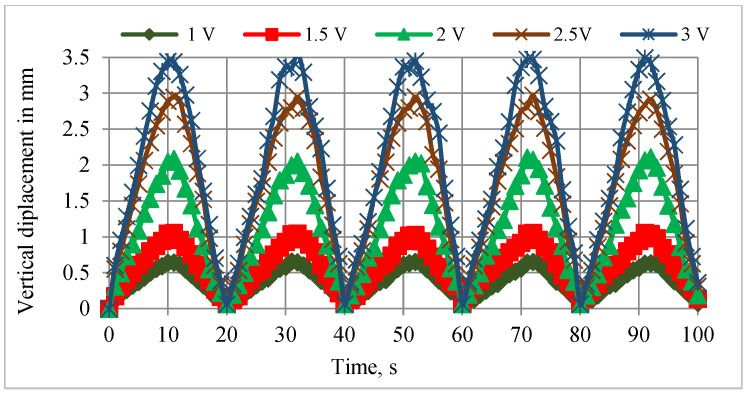
Vertical displacements for various inputs.

**Figure 8:**
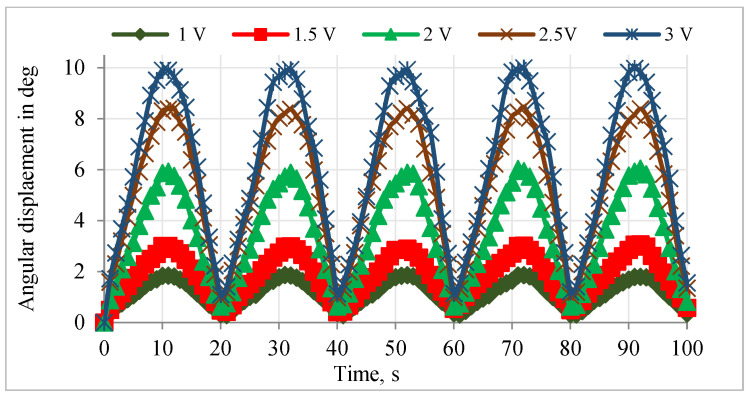
Angular displacements for various inputs.

**Figure 9:**
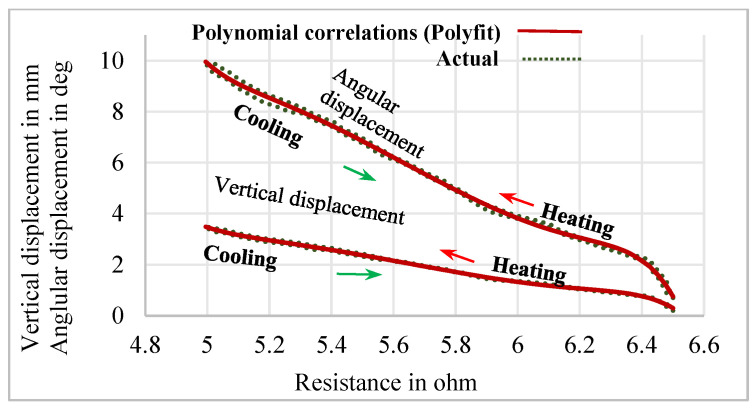
Relationship between displacements with respect to resistance.

The relationship between the vertical and angular displacements influenced by the resistance variation of SMPICs was investigated through experimentation results. The SMPICs were activated with a slow input frequency sine wave signal to obtain the maximum possible displacement. The tests were conducted under different magnitudes of input voltages (1 V, 1.5 V, 2 V, 2.5 V, and 3 V). The data on the SMPIC resistance variation, both the vertical and angular displacements of the mirror, were collected at the rate of 1 sample per second for a total time of 100 s (5 cycles) for a given sine wave input of 0.05 Hz frequency, as shown in Figure 6, Figure 7 and Figure 8. The relationship between the resistance of the SMPIC and the vertical displacement of the mirror, as well as the resistance of the SMPIC and the angular displacement of the mirror, are shown in Figure 9, and it reveals has no hysteresis. Hence, it was possible to obtain the vertical and angular displacements of the mirror by using the resistance changes of the SMPICs when actuated.

Using the Polyfit function in MATLAB^®^ 2019, two different polynomial correlations were obtained to estimate the vertical and angular displacements of the mirror using the resistance variation of the SMPIC. The seventh-order polynomial and polynomial correlation between the vertical displacement of the mirror and resistance of the SMPIC are given by the following equations:

(6) $$y_{Y}=-5.6x^{7}+{222.44x}^{6}-3786.86x^{5}+{35805.03x}^{4}-203061.08x^{3}\;+{\;\;690767.07x}^{2}-1305079.30x-1056429.35$$

(7) $$R_{Y}^{2}=-0.98993$$

The seventh-order polynomial and polynomial correlation between the angular displacement of the manipulator and the resistance of the SMPIC are given in Equations (8) and (9).

(8) $$y_{\theta }=-18.46x^{7}+{735.09x}^{6}-12540.81x^{5}+{118805.93x}^{4}-675000.08x^{3}\;+{2299960.54x}^{2}-4351711.72x-3527070.05$$

(9) $$R_{\theta }^{2}=-0.99606$$

**Figure 10:**
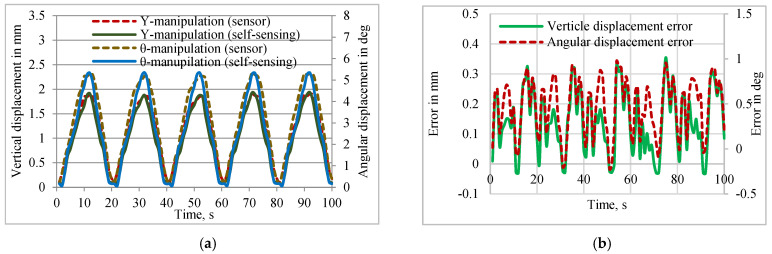
Validation of self-sensing with measured data using the external sensor. (**a**) Vertical and angular displacements. (**b**) Errors associated with displacements.

Figure 10a presents the validation of the self-sensing with measured data using the external sensor and the error associated with the vertical and angular displacements between the self-sensing and sensor-based measurements. Figure 10b reveals that the obtained seventh-order polynomial could estimate the mirror displacement using the resistance changes of the SMPIC with a minimum error compared to the displacements measured using a laser displacement sensor.

## 4. Design of Controller

About 3 decades ago in 1997, Grant and Hayward used Variable Structure Control to control the SMA actuators based on the switching operation. They actuated depending upon the sign of the error.

The control logic of the VSC controller is

$$if\;S>0\;U=V^{+},\;$$

$$if\;S<0\;U=V^{-}$$

$$S\;\mathrm{is}\;\mathrm{defined}\;\mathrm{as}\;S=Error\left(D_{d}-D_{a}\right)$$

where *D_a_* is the actual displacement, and *D_d_* is the desired displacement.

**Figure 11:**
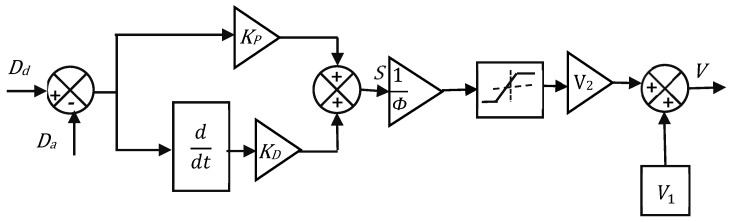
Block diagram of the PD-VSC control logic.

The objective requirement of the control system for an optimum response is an appropriate switching function design. Hence, the switching condition *S* is designed as the summation of both the weighted position error and the weighted velocity error, and it is named as the proportional derivative combined variable structure controller (PD-VSC). The block diagram of the control scheme is shown in Figure 11.

The control logic of the PD-VSC controller is as follows:

$$If,\frac{S}{\Phi }\geq +1V=V_{H},V_{H}=V_{1}+V_{2}$$

$$If,\frac{S}{\Phi }\leq -1V=V_{L},V_{L}=V_{1}-V_{2}$$

$$If,+1>\frac{S}{\Phi }<-1,V=K.S$$

In the above, V is the feedback control signal, *Φ* is the boundary layer thickness, and *S* is defined as

(10) $$S=K_{D}.\frac{d}{dt}\left(D_{d}-D_{a}\right)+K_{P}.\left(D_{d}-D_{a}\right)$$

where, *D_a_* is the displacement of the SMPIC actuator, *D_d_* is the desired displacement, $K_{D}\;$ is the weighted factor of velocity error (derivative gain), and $K_{P}$ is the weighted factor of the position error (proportional gain). The thickness of the boundary layer Φ is the region where this control law is applicable. The design parameters of the proposed controller are $V_{1}$, $V_{2}$, *Φ*, $K_{D}$, and $K_{P}$. Choose ${V_{1}\;and\;V_{2}\;according\;to\;V}_{1}+V_{2}=$ maximum actuating voltage and $V_{1}-V_{2}=$ minimum actuating voltage. In this case, $V_{1}$ = 1.75 V and $V_{2}$ = 1.25 V. The main advantage of *Φ* is that it minimized the chattering under steady state conditions. With the increasing value of *Φ*, the steady state error is reduced. Simultaneously, the control activity increased, thus resulting in a chattering response. With the decreasing value of *Φ*, the time control activity reduced, while the steady state error increased. Proportional gain increased the speed of the response. The derivative gain smoothened the control action without affecting the steady state condition. This was achieved while selecting the derivative gain $K_{D}$ close to less than one, which kept the angular velocity error as small as possible, thus reducing overshoot while ensuring a smooth control action.

### 4.1. Tuning of Control Parameters

*Step 1*: Consider the boundary layer Φ as unity and derivative gain $K_{D}$ as unity, and increase the proportional gain $K_{P}$ from 1 to obtain the response without overshooting.

*Step 2*: Consider the derivative gain $K_{D}$ as unity and the proportional gain $K_{P}\;$obtained from Step 1 as the optimum value of the boundary layer Φ to choose without any steady state error.

*Step 3*: Consider the proportional gain $K_{P}$ and the boundary layer Φ obtained from Steps 1 and 2; the derivative gain $K_{D}$ is decreased from 1 to ensure smooth control action with no steady state chattering.

Using the control parameters $K_{D},$ Φ, and $K_{P}\;$obtained in Steps 1, 2, and 3, the control performance is executed in MATLAB^®^ Simulink using the model obtained from the system identification while checking whether the errors (ISE and IAE) are within the limits. If the errors are within the limits, the control parameters are applied to experimental control for displacement control of the SMA/Polyimide composite actuator. If the errors are not within the limits, Steps 1, 2, and 3 are repeated using updated control parameters to obtain optimal values, i.e., the errors are within the limits. A flow chart for tuning the control parameters is presented in a subsequent section.

### 4.2. Control Strategy

Two PD-VSC controllers were designed for Y and θ manipulation control, and the control parameters of the controller were obtained analytically using the MATLAB^®^/SIMULINK by adapting the procedure according to the flowchart in Figure 10 using the model developed from the system identification in Equations (4) and (5). By using this control parameter, real-time experiments were conducted to examine the controlled operation of mirror manipulator with the PD-VSC.

#### 4.2.1. Control of Y Manipulation

**Figure 12:**
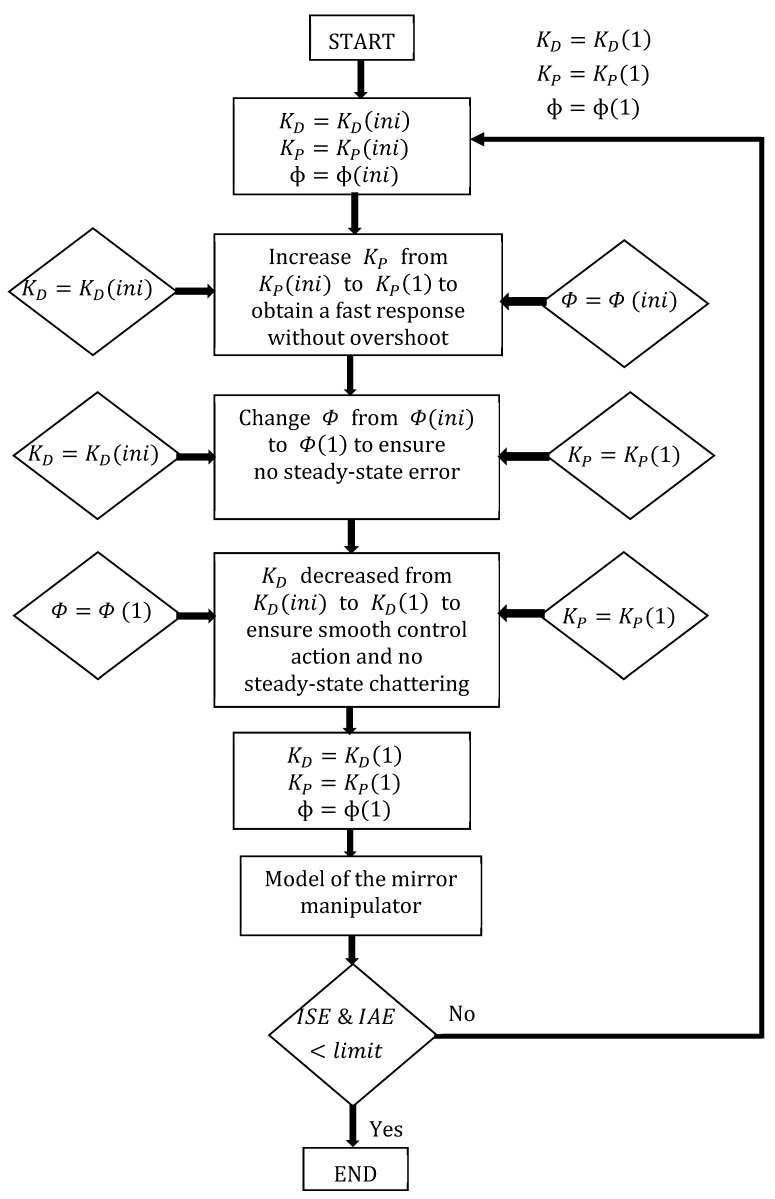
Flow chart for tuning the control parameters.

**Figure 13:**
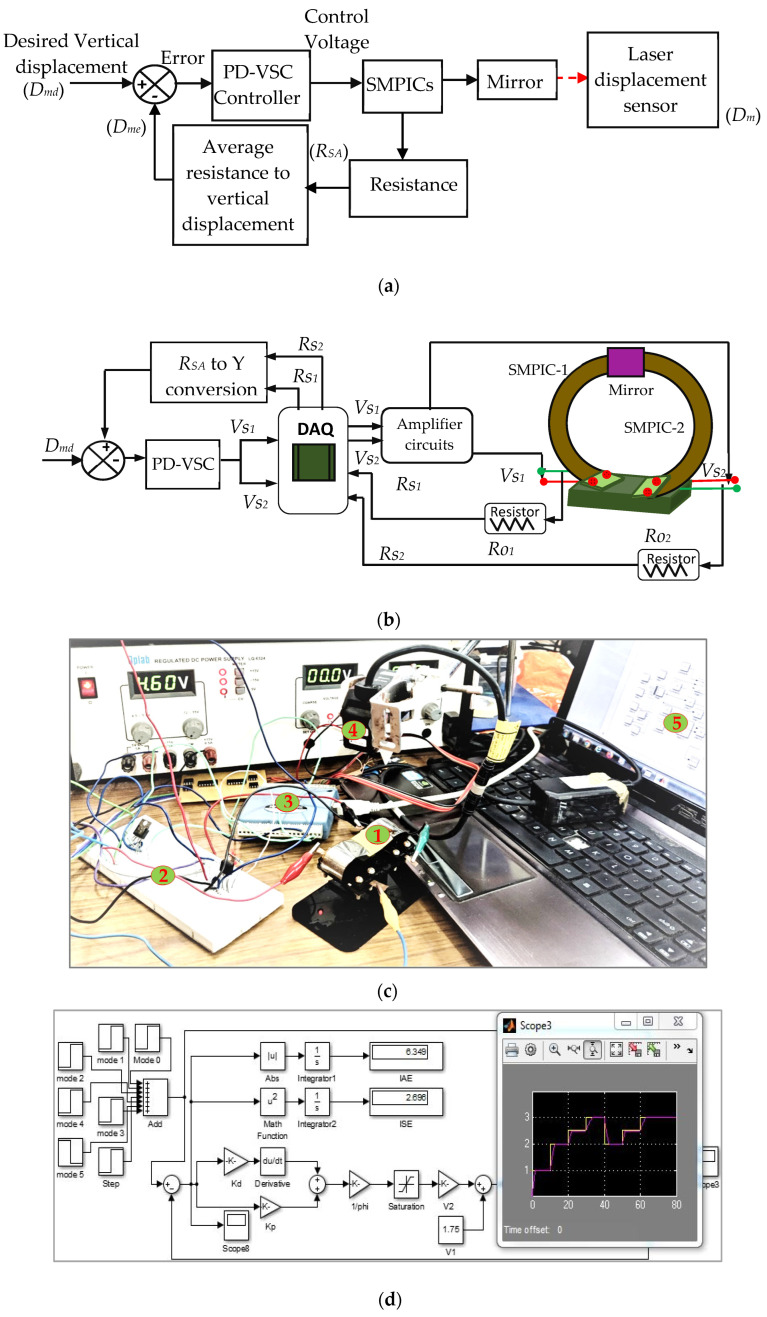
Control scheme of the SMPIC actuated and sensed mirror manipulator. (**a**) Block diagram. (**b**) Schematic representation. (**c**) Photograph: 1. Mirror manipulator, 2. Amplifier circuits, 3. DAQ, 4. Laser Displacement Sensor, 5. Control circuit in MATLAB^®^. (**d**) MATLAB^®^ simulation block for control of the mirror manipulator.

To implement the sensor-less control of the mirror manipulator, the proportional derivative combined variable structure controller was used based on the flow chart shown in Figure 12. For Y manipulation control, the desired input (*D_md_*) was given to the PD-VSC and control voltage sent to both of the SMPICs through DAQ and amplifier circuits. Due to the actuation of both SMPICs, the mirror moves vertically up in direction (*D_m_*), while the resistance changes of both SMPICs (*R_S_*_1_ and *R_S_*_2_) are measured. The average value of the measured resistance *R_SA_* (average of *R_S_*_1_ and *R_S_*_2_) was converted into vertical displacement (*D_me_*) using the polynomial correlation function given in Equation (6), and this was compared with the desired input (*D_md_*). Finally, the estimated position and velocity error was sent to the PD-VSC controller. Based on the error value, the controller provides the control voltage to reach the desired input. The block diagram, schematic representation, and photograph of the Y manipulation control is shown in Figure 13a,b. A photograph of the experimentation and the MATLAB^®^ simulation block for the control of the mirror manipulator are shown in Figure 13c,d. In the figure, *D_md_* is the desired input for the vertical displacement in mm; *D_m_* is the vertical displacement measured by LDS in mm; *D_me_* is the vertical displacement estimated from the Polyfit function in mm; and *R_SA_* is average resistance of *R_S_*_1_ and *R_S_*_2_ in ohms.

#### 4.2.2. Control of θ Manipulation

**Figure 14:**
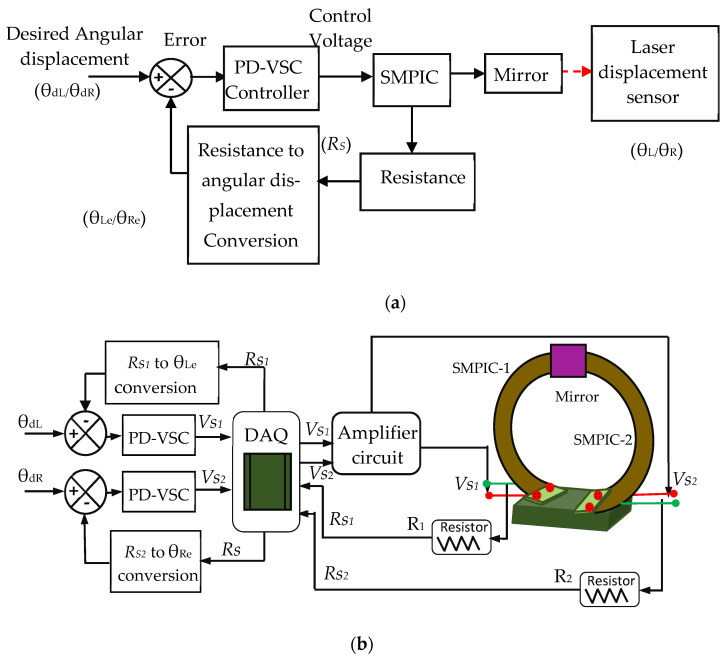
Block diagram and schematic representation of θ manipulation control. (**a**) Block diagram. (**b**) Schematic representation.

For θ manipulation control, the desired inputs (θ_dL_ or θ_dR_) were given to the PD-VSC, while the control voltage was sent to any one of the SMPICs (SMPIC-1 for θ_L_ and SMPIC-1 for θ_R_) through the corresponding DAQ and amplifier circuits. Due to the actuation of either of the SMPICs, the mirror moved in the corresponding angular direction, and the resistance changes of the corresponding SMPIC were measured. The measured resistance was converted into angular displacement using the polynomial correlation function given in Equation (8). This angular displacement was compared with the desired input. Finally, the estimated position and velocity error was sent to the PD-VSC controller, thus depending on the error value the controller gave the control voltage to reach the desired input. The block diagram and schematic representation of the θ manipulation control are shown in Figure 14a,b, respectively. In these figures, θ_dL_ is the desired input for the left angular displacement in deg; θ_L_ is the left angular displacement measured by LDS in deg; θ_Le_ is the left angular displacement estimated from the Polyfit function in deg; θ_dR_ is the desired input for the right angular displacement in deg; θ_R_ is the right angular displacement measured by LDS in deg; and θ_Re_ is the right angular displacement estimated from the Polyfit function in deg.

## 5. Results and Discussion

### 5.1. Comparison of Performances with and without Control

**Figure 15:**
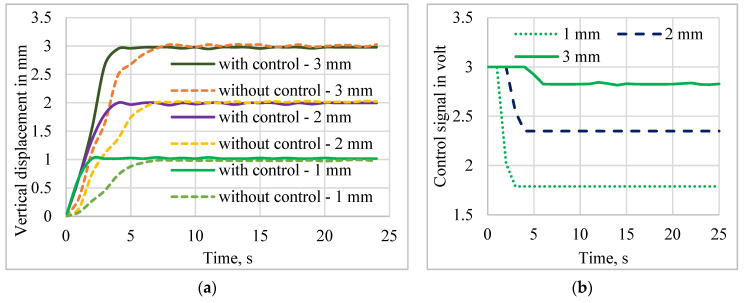
Performance comparison of the open-loop and closed-loop using self-sensing PD-VSC. (**a**) Vertical displacement. (**b**) Control voltage.

Sensor-less control of the mirror manipulator was implemented, and the results are presented to prove the effectiveness of the self-sensing-based closed-loop system with the proposed PD-VSC control technique. The applied voltage was limited to 3 V, as well as the safe heating value, to prevent overheating of the SMPICs. The real-time system performances for the tracking and disturbance rejection were investigated for pulse, sine, and multistep tracking signals. The response of the sensor-less control was compared with the open-loop system performances, and the closed-loop control of the mirror position in the vertical direction for the desired inputs of 1 mm, 2 mm, and 3 mm was executed experimentally. The controlled mirror displacement output and control voltage input concerning time were measured. The input to the open-loop system (without control) taken from the control signal’s final settling time voltage was given to the open loop system, which measured the displacement for the without control system. The responses of the without control and with control systems are shown in Figure 15. The control voltage of the closed-loop system is shown in Figure 15b. It has been clearly observed that the mirror manipulator control using the PD-VSC could track the desired input signal faster than the without control method. The settling time increased with increased displacements. For minimum manipulation, a faster response was obtained, and for maximum manipulation, a slow response was obtained. Hence, based upon the requirement, the fast response or maximum displacements can be chosen as a principal control target. Also, compared to other SMA actuators, the proposed structure has a better cooling response due to the bias force given by the trained polyimide substrate and the weight of the mirror.

### 5.2. Control Performances

#### 5.2.1. Tracking Response of Pulse Trajectory

**Figure 16:**
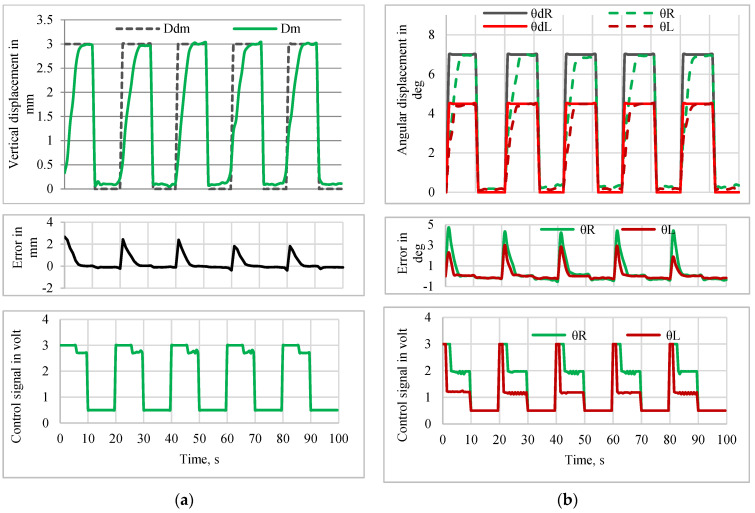
Tracking performance for pulse input. (**a**) Y-manipulation control. (**b**) θ-manipulation control.

The tracking performance of the manipulator using the PD-VSC control system for tracking with a pulse, sine wave, and multistep inputs was experimentally verified. A pulse trajectory of amplitude 3 mm with a 50% duty cycle having an action period of 20 s was given as the desired input (*D_dm_*) to a sensor-less closed-loop control system for the Y manipulation control of the mirror manipulator. The control action was executed in MATLAB^®^, and the controlled voltage developed by the PD-VSC controller was the input to both SMPICs that measured the corresponding vertical displacement (*D_m_*). Figure 16a shows the position tracking performance, error, and respective control action for the Y manipulation control. The position tracking performance, error, and respective control action for the θ manipulation control, while tracking a pulse trajectory of amplitude of 7 deg for the right angle direction (*θ_R_*) and 5 deg for the left angle direction (*θ_L_*) with a 50% duty cycle and an action period of 20 s, are as shown in Figure 16b.

#### 5.2.2. Tracking Response of Sine and Multistep Trajectory

**Figure 17:**
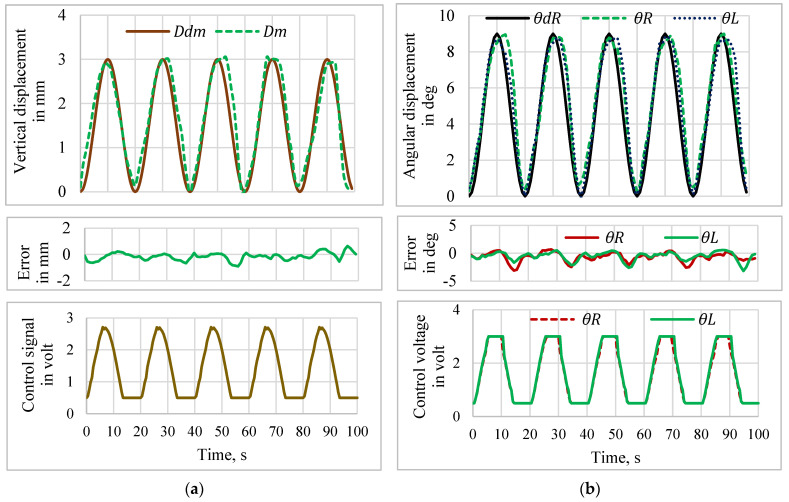
Tracking performance for the sine input. (**a**) Y-manipulation control. (**b**) θ-manipulation control.

**Figure 18:**
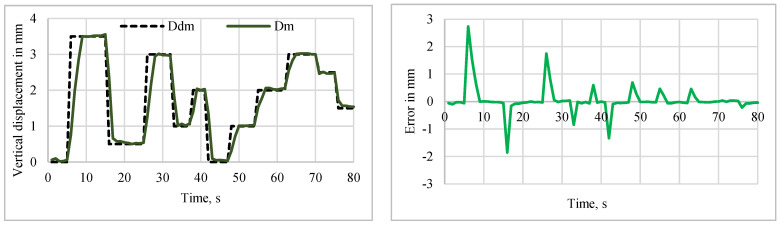
Tracking performance of the Y manipulation with multistep inputs.

**Figure 19:**
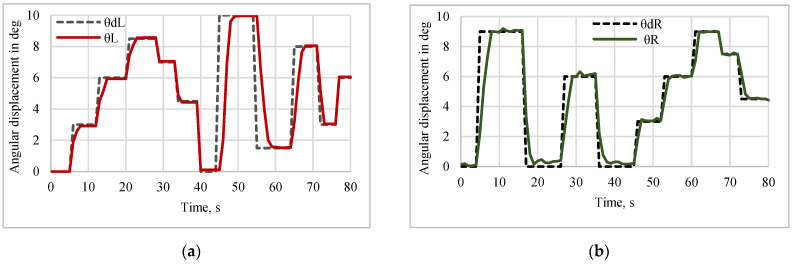
Tracking performance of θ manipulation (θ-Left) with multistep inputs. (**a**) Left angle displacement. (**b**) Right angle displacement.

The position tracking performance, error, and respective control action for the Y manipulation control, while tracking a sine trajectory of amplitude 3 mm with a 50% duty cycle and an action period of 20 s, are shown in Figure 17a. The position tracking performance, error, and respective control action for the θ manipulation control, while tracking a sine trajectory of amplitude 9 deg for the right angle direction (θ_R_) and the left angle direction (θ_L_) with a 50% duty cycle with an action period of 20 s, are shown in Figure 17b. The position tracking performance and error for the Y manipulation control, while tracking a multistep trajectory for different amplitudes for an action period of 80 s, are shown in Figure 18. The position tracking performance of the θ manipulation control, while tracking a multistep trajectory for different amplitudes for an action period of 80 s, is shown in Figure 19. Figure 15, Figure 16, Figure 17, Figure 18 and Figure 19 reveal that the error associated with controlled output response for the step, pulse, sine and multistep trajectories of the mirror manipulator was at a minimum when the desired change in input was at a minimum, i.e., without a high magnitude change, and it was at a maximum when the desired change in input was at a maximum, i.e., with a high magnitude change. Hence, the implemented control technic for SMPICs is most suitable, where the change in input is at a minimum. The position tracking performance of the θ manipulation control for the teft angle direction (θ_L_) and the right angle direction (θ_R_) while tracking a multistep trajectory for different amplitudes for action periods of 80 s is shown in Figure 18.

#### 5.2.3. Disturbance Rejection

**Figure 20:**
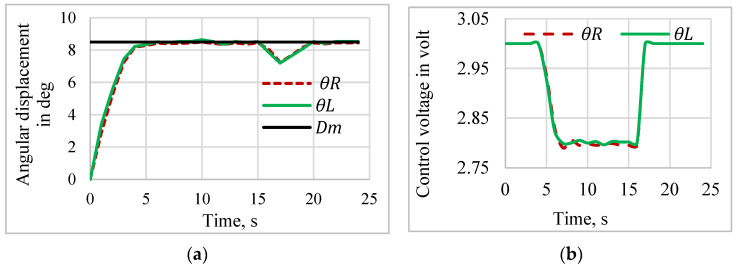
Disturbance rejection response of the sensorless control system. (**a**) Disturbance rejection response. (**b**) Control voltage.

The disturbance rejection performance was evaluated for the left- and right-angle displacements. The desired step signal with an 8.5 deg amplitude was given to the proposed controller with a disturbance of a 50 mg load added to the bottom of the mirror after 15 s of actuation. Figure 20 shows the disturbance rejection behavior and the respective control action. The proposed controller showed high accuracy and smooth control action in the disturbance rejection activities. According to the proposed self-sensing-based PD-VSC’s closed-loop performances, depicted in Figure 14, Figure 15, Figure 16, Figure 17, Figure 18, Figure 19 and Figure 20, the PD-VSC could track the position and overcome the disturbances faster with no overshoot and without a steady state error.

## 6. Conclusions

In this work, a mirror manipulator was designed using shape memory polyimide composites (SMPICs) actuated using joule heating, and we verified the vertical and angular displacements (Y/θ manipulations) for various input voltages and frequencies. The open-loop testing (Y and θ manipulations) and the relationship between the resistances of the SMPICs and the vertical and angular displacement of the manipulator were examined. The SMPICs underwent resistance variations from 6.5 ohms to 4.99 ohms for the vertical displacement changes from 0 mm to 3.5 mm, while the angular displacement changed from 0 mm to 9.88 deg. Polynomial correlations between the resistances with the displacements of the mirror manipulator were developed using Polyfit functions, which were employed to estimate the vertical and angular displacements of the mirror manipulator by measuring the resistance variations of the SMPIC actuators. It is evident from the results of the polynomial correlation that an accurate estimation of the vertical and angular displacements of the mirror manipulator can be achieved. A proportional derivative controller combined with a variable structure controller (PD-VSC) was formulated and experimentally realized to obtain accurate tracking of the sinusoidal and multistep trajectories. The one salient feature of the proposed control system is the sensor-less control of the mirror’s position in the vertical and angular directions, thus resulting in the cost-effectiveness in the closed-loop control system. It is finally noted that the control method and experimental results presented in this work are quite self-explanatory, thus justifying that shape memory can be widely used as a soft actuator, especially for soft robotic and soft wearable applications.

## Figures and Tables

**Table 1:** Dimensions of the mirror manipulator.

Property	Value	
	SMPICs 1 and 2	Mirror
Thickness	76.75 μm (Kapton—75 μm + SMA—1.75 μm)	100 μm
Length	50 mm	30 mm
Width	20 mm	20 mm

**Table 2:** Displacement of the mirror manipulator for various input voltages.

Input Voltage (V)	*D_m_* (mm)	*θ_L_* (deg)	*θ_R_* (deg)	*R_S_*_1_ (ohm)	*R_S_*_2_ (ohm)
0.00	0.00	0.00	0.00	6.50	6.50
1.00	0.32	1.15	0.94	6.49	6.48
1.50	0.62	1.80	1.71	6.44	6.40
2.00	1.49	4.68	4.20	5.92	5.87
2.25	1.78	6.07	5.72	5.76	5.70
2.50	2.42	7.97	7.75	5.43	5.44
2.75	2.90	9.33	9.10	5.18	5.16
3.00	3.37	10.84	9.95	4.99	5.01

**Table 3:** Displacement of the mirror manipulator for various input frequencies.

Input Frequency (Hz)	*D_m_* (mm)	*θ_L_* (deg)	*θ_R_* (deg)	*R_S_*_1_ (ohm)	*R_S_*_2_ (ohm)
0.05	3.39	9.75	9.72	4.98	4.98
0.10	3.21	9.26	9.21	5.09	5.08
0.20	2.56	7.28	7.36	5.37	5.35
0.33	1.70	4.63	4.78	5.78	5.77
0.50	0.99	2.67	2.66	6.22	6.20

## Data Availability

The original contributions presented in the study are included in the article, further inquiries can be directed to the corresponding author.
